# Physiology and transcriptome of *Eucommia ulmoides* seeds at different germination stages

**DOI:** 10.1080/15592324.2024.2329487

**Published:** 2024-03-17

**Authors:** Jia Liu, Sumei Qiu, Tingting Xue, Yingdan Yuan

**Affiliations:** a Department of Civil and Architecture and Engineering, Chuzhou University, Chuzhou, Anhui, China; b Anhui Low Carbon Highway Engineering Research Center, Chuzhou University, Anhui, China; c College of Horticulture and Landscape Architecture, Yangzhou University, Yangzhou, China

**Keywords:** *Eucommia ulmoides*, hormone content, seed germination, transcriptome

## Abstract

*E. ulmoides (Eucommia ulmoides)* has significant industrial and medicinal value and high market demand. *E. ulmoides* grows seedlings through sowing. According to previous studies, plant hormones have been shown to regulate seed germination. To understand the relationship between hormones and *E. ulmoides* seed germination, we focused on examining the changes in various indicators during the germination stage of *E. ulmoides* seeds. We measured the levels of physiological and hormone indicators in *E. ulmoides* seeds at different germination stages and found that the levels of abscisic acid (ABA), gibberellin (GA), and indole acetic acid (IAA) significantly varied as the seeds germinated. Furthermore, we confirmed that ABA, GA, and IAA are essential hormones in the germination of *E. ulmoides* seeds using Gene Ontology and Kyoto Encyclopedia of Genes and Genomics enrichment analyses of the transcriptome. The discovery of hormone-related synthesis pathways in the control group of Eucommia seeds at different germination stages further confirmed this conclusion. This study provides a basis for further research into the regulatory mechanisms of *E. ulmoides* seeds at different germination stages and the relationship between other seed germination and plant hormones.

## 1. Introduction

*E. ulmoides (Eucommia ulmoides)*, also known as bakelite, is a member of the genus *Eucommia* and family *Eucommiaceae*.^1^ *E. ulmoides* is highly adaptable and prefers a sunny, cool, and warm environment.^2^ *E. ulmoides*, a paleontological species with an ancient origin, was left behind by glacial movements in the third century.^3^ In addition to its ornamental value, *E. ulmoides* has a very high therapeutic value and is an excellent raw material for producing foods, medicines, and healthcare products. Furthermore, *E. ulmoides* also has significant industrial importance and is the best natural rubber alternative.^4,5^ The demand for *E. ulmoides* has gradually increased in the medicinal and industrial markets, exacerbating the scarcity of *E. ulmoides* resources. Therefore, it is crucial to breed *E. ulmoides* seedlings.^2,6^ Sowing, cutting, and root tillering seedlings are standard methods to propagate *E. ulmoides*. Although several breeding methods exist, sowing seedlings is a standard production method. This is because seedlings grown from sowing develop root systems, strong stress resistance, and long lifespans.^7,8^ However, most of the research on *E. ulmoides* has focused on its medicinal and industrial value, and there are not many studies on the germination of *E. ulmoides* seeds.

Seed germination is a prerequisite for normal plant growth and development, a crucial stage in the continuation and development of seed plant races, and is of great biological significance.^8^ Previous studies have found that the plant hormones abscisic acid (ABA), indole acetic acid (IAA), jasmonic acid (JA), gibberellin (GA), and salicylic acid (SA) are related to seed germination.^9–11^ ABA can inhibit seed germination,^12^ but the balance between different hormones will significantly affect seed germination and is related to changes in the absolute concentration of specific hormones.^13^ GA in plants can actively promote seed germination by lowering the inhibitory effect of ABA.^14,15^ In addition, several endogenous hormones, including IAA, JA and SA, directly or indirectly participate in the ABA or GA signaling pathway to regulate seed germination.^16^ Several studies have shown ABA, IAA, and GA play a major regulatory role in seed germination.^17,18^ Changes in the proportion of different hormones are critical in seed germination relative to a single endogenous hormone.^19,20^ Seed germination is regulated by the dynamic balance of ABA and GA contents.^21^ For example, the decreased expression of CYP707A2 increases the CSP2 protein levels, which has a cold shock domain. At this point, a decrease in GA content results from downregulating the GA synthesis genes GA20ox and GA30ox. Therefore, seeds with overexpressed CSP2 have lower germination potential,^22^ demonstrating the dynamic balance between these two hormones during seed germination. IAA positively regulates ABA and negatively regulates GA biosynthesis and signal transduction pathways during seed germination.^18,23,24^ IAA and ABA signaling are cross-regulated, influencing seed germination.^25^ IAA inhibits seed germination in high salt-stress environments.^26^ Simultaneously, ABA inhibits auxin signaling, prevents hypocotyl extension, and further inhibits seed germination.^27^

The study of transcriptomics is to collect all RNA expressed in a specific cell or tissue at a specific developmental period, catalog coding RNA and non-coding RNA, thereby determining the transcription structure and function of genes, and revealing the mechanism of action of related regulatory genes.^28^ Compared with traditional technical methods, RNA-seq has the characteristics of high throughput, good accuracy, and high reproducibility. It has become an efficient method for gene expression research and is widely used. Zaynab conducted transcriptome sequencing analysis between germinated and ungerminated seeds of Trichosanthes kris, and found that differential genes were mainly enriched in glycolysis, starch and sugar metabolism, phytohormone signaling, carbohydrate metabolism, and ribosome structure and biology.^29^ Liu et al. studied the transcriptional dynamics of embryonic leaves during corn seed germination, identified differentially expressed genes at different time periods, and analyzed the gene function classification of these differentially expressed genes, thereby revealing the developmental processes of embryonic leaves 72 hours before corn seed germination. Dynamic curves of transcriptional and physiological transitions.^30,31^ Chen et al. analyzed the transcriptome data of wheat varieties with different dormancy phenotypes and found five genes TaC3H4/-18/-37/-51/-72 that may be involved in wheat seed dormancy and germination. In order to reveal wheat C3H zinc finger transcription It provides a reference for the role of factors in biotic and abiotic stresses in different seeds of Chrysanthemum vulgaris.^32^ It can be seen that transcriptome sequencing technology has set off an upsurge in the field of seed germination research. This study intends to use this technology to reveal the molecular mechanism of *E. ulmoides* seed germination.

The ability of plants to survive and adapt to environmental changes depends on seed germination.^33^ Therefore, gaining insight into the dynamic changes in endogenous hormone levels, understanding the effects of different endogenous hormones *Eucommia ulmoides* seeds germination and revealing the relationship between them is of great significance to promoting the cultivation and utilization of *E. ulmoides*. It also provides a basis for further research on the effects of related plant hormones on seed germination. The impact provides a theoretical basis. This study used dry seeds with the peel removed that absorbed water for 2 days, seeds with the peel removed that absorbed water for 4 days, seeds with scratched endosperm that continued to cultivate for 12 hours, and newly germinated seeds with scratched endosperm to understand the morphological structure and physiological indicators of *E. ulmoides* seeds at different germination stages. Scientific analysis is conducted to find the differential genes in the germination process of *E. ulmoides* seeds, which provides important scientific basis for the research on *E. ulmoides* seed germination.

## 2. Materials and methods

### 2.1. Plant material and sample collection

The *E. ulmoides* seeds used in this study were collected from Fengle Avenue, Chuzhou. The seeds were stored at room temperature in the Plant Physiology Laboratory of Chuzhou University. *Eucommia* seeds were soaked at room temperature for 10 days before being placed in a germination box covered with moist absorbent cotton and kept in an incubator overnight at a constant temperature of 30°C. Five samples were collected during the germination process of the seeds: DZ1 (dried seeds, peeled), DZ2 (peeled seeds absorbed water for 2 days), DZ3 (peeled seeds absorb water for 4 days), DZ4 (seeds continued to cultivate for 12 hours after cutting the endosperm), and DZ5 (newly germinated seeds after cutting the endosperm). At each germination stage, we collected 5 to 10 seeds at the same stage, disinfected their surfaces with 20% sodium hypochlorite for 4 min, and then rinsed them five times with MilliQ water.

### 2.2. Determination of physiological indicators and hormone contents

The anthrone sulfate method calculated the total soluble sugar content^34^; The cellulose content was determined colorimetrically using the blue-green reaction of the anthrone reagent and furfural compounds. The amylose and amylopectin content were determined using spectrophotometry. Cellulase content was determined using the anthrone colorimetric method. Yang et al. described the method to determine the hormone content.^35^ Endogenous hormones were extracted, and the competitive enzyme-linked immunoassay method was used to determine the ABA, IAA, JA, GA3, and SA hormone levels in *E. ulmoides* seeds at different germination stages.

### 2.3. Morphological structure observation

We took a piece of the peel and endosperm surface from a *E. ulmoides* seed, fixed them on the sample using a carbon double-sided tape, adjusted the height of the sample stage, placed it in the sample chamber, and then observed it under a scanning electron microscope (S-4800II, Hitachi, Japan). We used a transmission electron microscope to observe and HITACHI-600 to capture images. After mounting the sample, the microstructure was analyzed using the conventional paraffin sectioning method, stained with safranin-fast green, and panoramic scans were performed on the Pannoramic250/MIDI scanner (3D HISTECH, Hungary).

### 2.4. Differentially expressed genes (DEGs) analysis of transcriptome data

DEGs can determine changes in gene expression under different experimental conditions, and each comparison results in a differential gene set. Studying the distribution of differential genes in Gene Ontology (GO) and the Kyoto Encyclopedia of Genes and Genomes (KEGG) can help understand their functions. GO enrichment analysis was carried out by annotating differentially expressed genes using the Gene Ontology (GO) database. KEGG pathway analyses of DEGs were performed for pathway enrichment analysis using the Kyoto Encyclopedia of Genes and Genomes (KEGG) database. The three seed stages – DZ2, DZ3, and DZ5—of the *E. ulmoides* germination process were used as the sequencing material for transcriptome analysis. The |log_2_-Fold Change| ≥ 1 and false discovery rate (FDR) < 0.05 was used as the threshold to assess the significance of differential gene expression. GO is annotated and visualized using the clusterProfiler package, and the KEGG data is obtained from the EggNOG-mapper results and visualized in clusterProfiler.

### 2.5. Quantitative real-time polymerase chain reaction (qRT-PCR) analysis

Nine representatives of differently expressed genes were selected, and their expression levels were detected by timed qRT-PCR. The remaining total RNA from transcriptome sequencing was used for qRT-PCR analysis. The relative expression levels of the selected DEGs normalized to an internal reference gene Actin was calculated using 2^−ΔΔCt^ method.^36^ The specific primers for DEGs were designed by Oligo 7 software. The qRT-PCR was performed on an ABI 7500 Real-time PCR system (Applied Biosystems, USA) using SYBR Premix Ex Taq (Takara, Japan) according to the manufacturer’s protocol.

## 3. Results

### 3.1. Changes in plant hormone content and physiological indicators at different germination stages

**Figure 1.**
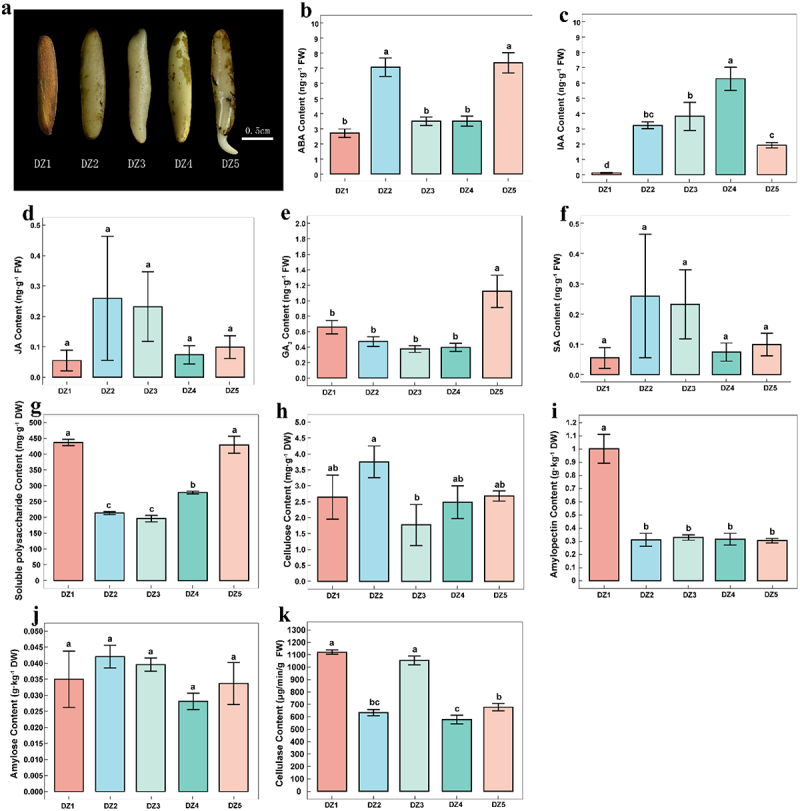
(a) Morphological characteristics of *Eucommia ulmoides* at different germination stages. (b) Abscisic acid (ABA) content; (c) Indole acetic acid (IAA) content; (d) Jasmonic acid (JA) content; (e) Gibberellin (GA_3_) content; (f) Salicylic acid (SA) content; (g) Soluble polysaccharide content; (h) Cellulose content; (i) Amylopectin content; (j) Amylose content; and (k) Cellulase content.

This study determined ABA, auxin, IAA, JA, GA3, and SA levels at five germination stages (Figure 1a). First, DZ2 and DZ5 had relatively high levels of ABA, significantly different from the other three stages (Figure 1b). During the seed germination process, the IAA level showed an initial trend of increasing and decreasing, reaching its highest value (6.28 ng g^−1^ FW) in the DZ4 stage. The variance analysis findings demonstrated significant differences between the four stages of DZ1, DZ3, DZ4, and DZ5 (Figure 1c). The GA content showed a trend of slowly decreasing and then increasing during the germination process of *E. ulmoides* seeds. It increased sharply from DZ4 to DZ5, reaching its maximum concentration in DZ5 (1.12 ng·g^−1^ FW), significantly different from the other four germination stages. (Figure 1e). However, there was no significant difference between JA and SA during the germination of *E. ulmoides* seeds (Figure 1d–f). As shown above, ABA, IAA, and GA regulate how *E. ulmoides* seeds germinate. Additionally, comparing the concentrations of different hormones in the late stage of germination with the early stage, it can be shown that ABA and GA account for a higher proportion in the later stage. In contrast, the content of IAA rapidly decreases in the later stage. Therefore, ABA, GA, and IAA are essential hormones for seed germination.

At various germination stages, we measured the physiological index content of *E. ulmoides* (Figure 1g–k). As shown in Figure 1g, DZ5 significantly differs from DZ2 and DZ3 in that it has the highest soluble polysaccharide content at 429.1 mg·g^−1^ DW. The highest cellulose content is found in DZ2 and the lowest in DZ3, as shown in Figure 1h. Between these two stages, there are significant differences. DZ1 contains the highest amylopectin content and then decreases sharply. The amylopectin content is only 3.11 g·kg^−1^ DW by the DZ2 period. The amylopectin content barely changed from the DZ2 to the DZ5 period and the difference is insignificant, however, amylopectin significantly differs between the DZ1 and the other four stages (Figure 1i). During the germination process of *E. ulmoides* seeds, the amylose content tended to increase, decrease, and then increase, with no significant difference (Figure 1j). In the DZ1 and DZ3 stages, the cellulase content is relatively high. Although the difference between DZ1 and DZ3 is insignificant, it differs significantly from the DZ4 and DZ5 stages, respectively (Figure 1k). According to the results above, soluble polysaccharides, cellulose, cellulase, and amylopectin of the *E. ulmoides* seeds will significantly differ depending on the different germination states. Still, the amylose content has no significant difference.

### 3.2. Morphological and cytological observation of seeds

**Figure 2.**
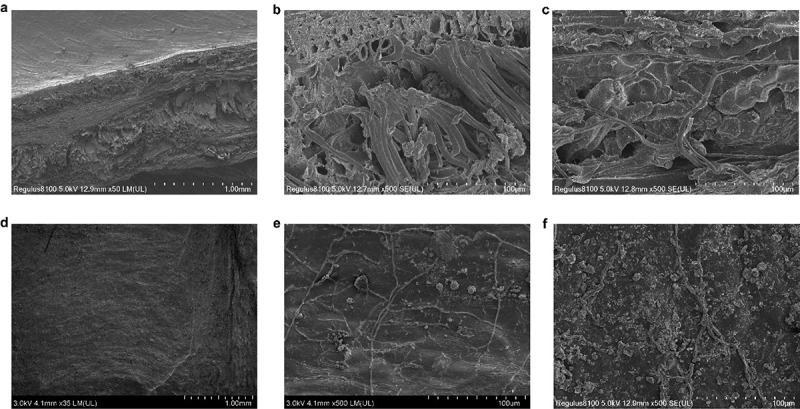
Scanning electron microscope image of *Eucommia ulmoides* seeds. (a-c) Peel section; (d-f) Endosperm surface layer.

**Figure 3.**
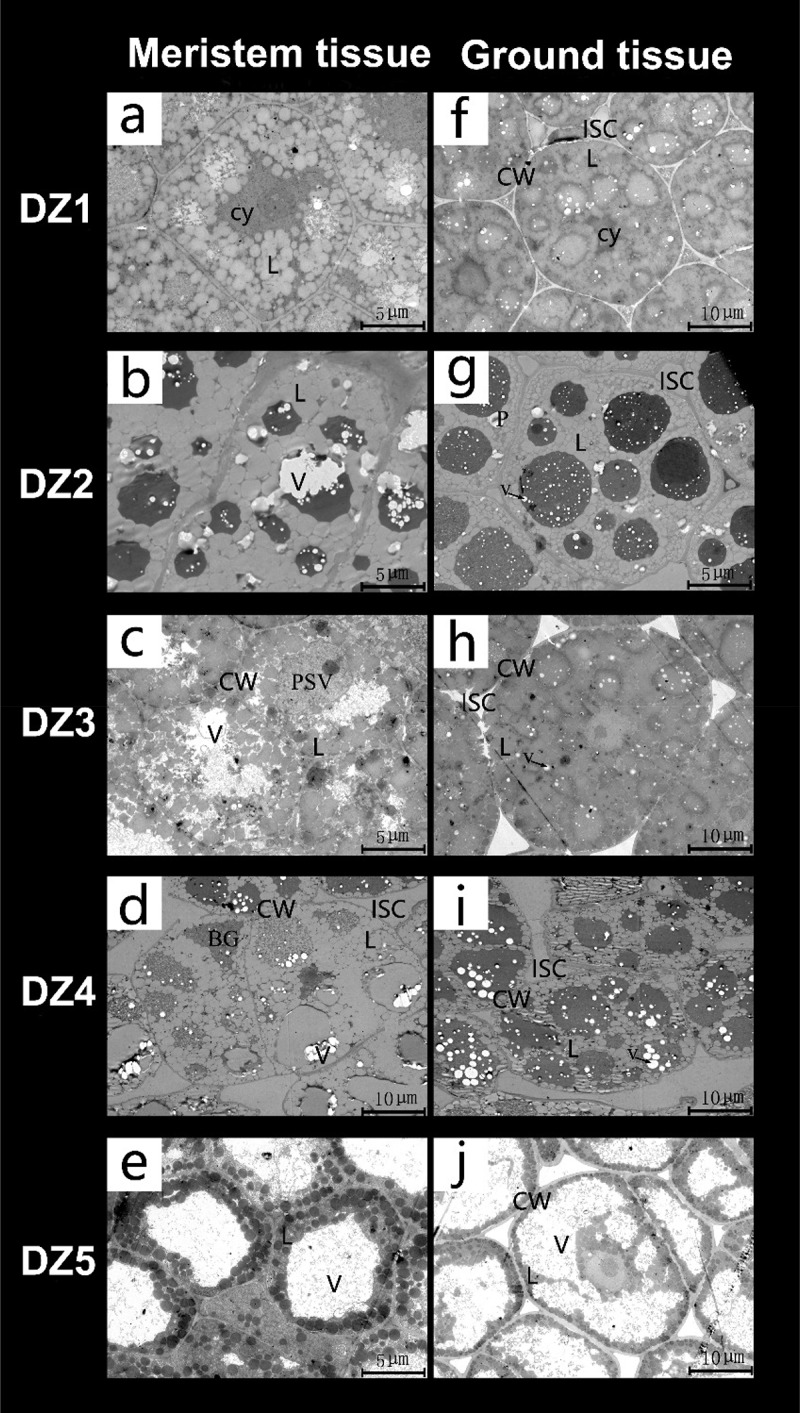
Transmission electron microscope images of the meristem and basic tissues in *Eucommia ulmoides* seeds at different germination stages.

We used SEM technology to examine the peel cross-section (Figure 2a–c) and endosperm surface layer (Figure 2d–f) of *E. ulmoides* seeds. Palisade cells and parenchyma cells were observed on the peel section. The surface of the endosperm is not smooth; it contains many small particles with clear veins. We used TEM technology to examine the ultrastructure of the meristem and fundamental tissues of *E. ulmoides* seeds at different germination stages. We discovered many high electron-density substances in the meristem and basic tissues of *E. ulmoides* seeds that had absorbed water for 2 days (Figure 3a, b). By the DZ3 stage, we observed that the amount of high electron density substances in the meristem and fundamental tissues had decreased from the DZ2 stage, but some of them remained. The difference is that the cell wall in the meristem disappears, and the water content increases, leaving gaps between cells in the fundamental tissue (Figure 3c). When the meristem divides to produce cells by the DZ4 stage, the cells are observed to be tightly arranged, with no intercellular gaps and dense cytoplasm. The vacuoles increase, and the cytoplasm in the fundamental tissue becomes thin (Figure 3d). At the DZ5 stage, when the radicle has just begun to extend out of the endosperm, many lipid droplets gather around the meristem cell walls, and the cell walls of the fundamental tissues become thin and increase in volume.

**Figure 4.**
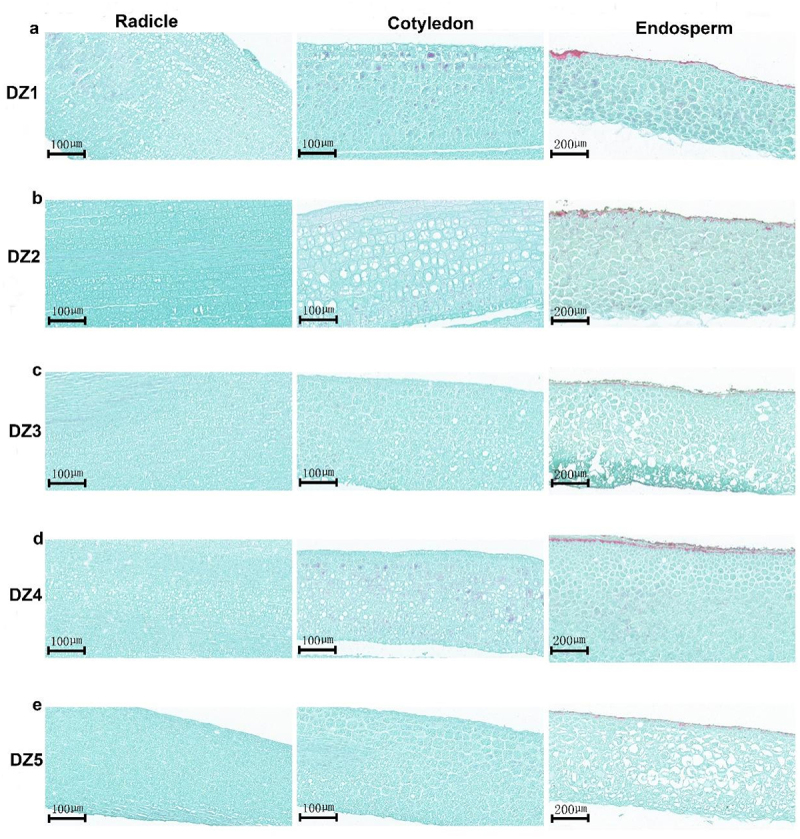
Paraffin sections of cell changes in the radicle, cotyledons, and endosperm of *Eucommia ulmoides* seeds at different germination stages.

We made paraffin sections of the radicle, cotyledon, and endosperm cell changes as the *E. ulmoides* seeds germinated (Figure 4). According to the observational findings, the cells are tightly and neatly arranged during the DZ2 stage of the radicle cell changes (Figure 4a,b). Endosperm cells begin to expand, starch granules multiply, and the cellular endosperm reaches its mature stage as the embryo develops (Figure 4c–e).

### 3.3. DEGs identification

**Figure 5.**
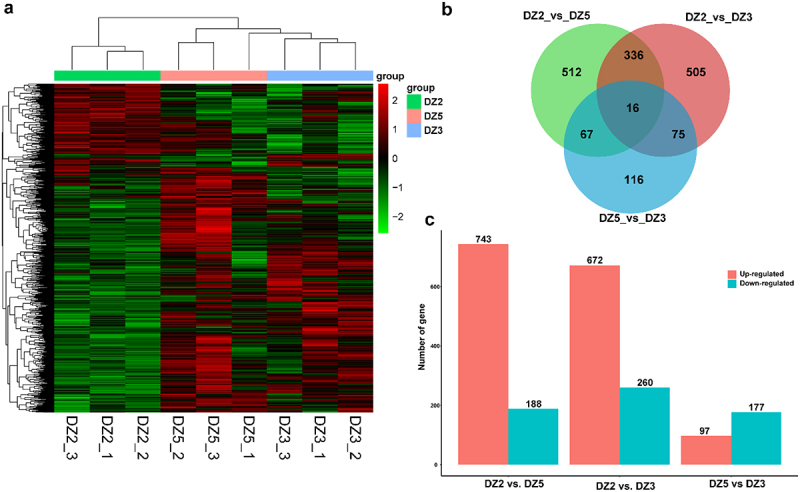
Analysis of differentially expressed genes during the germination stages of *Eucommia ulmoides*. (a)Gene expression of *Eucommia ulmoides* seeds at different germination stages; (b)A Venn diagram of gene expression. The green circle represents DZ2 vs. DZ5, the red circle represents DZ2 vs. DZ3, and the blue circle represents DZ5 vs. DZ3; (c)A column diagram of gene expression distribution in samples. Red represents significantly upregulated genes, and blue represents significantly downregulated genes.

According to biological characteristics and physiological experimental results, this study was divided into three different change stages, and the analysis was carried out in the three stages of DZ2, DZ3, and DZ5. The genes at different stages of the germination process of *E. ulmoides* seeds are analyzed using three biological repetitions as the standard. A differential expression analysis of gene expression was performed to obtain the expression of genes at different stages to understand the pattern of gene change during the seed germination of *E. ulmoides* (Figure 5a).

Using pairwise comparisons, a Venn diagram was used to compare the differential genes between the different stages of *E. ulmoides* seeds (Figure 5b). DZ2 and DZ5 share 91 differential genes, DZ3 shares 841 differential genes, and DZ2 expresses 183 differential genes. DZ2 and DZ3 share 83 differential genes, and DZ3 shares 191. DZ2 shares 848 differential genes with a medium level of expression. DZ5 and DZ3 share 352 differential genes, DZ3 shares 580 differential genes, and DZ5 shares 579 differential genes. DZ2, DZ3, and DZ5 have a total of 16 differential genes. *E. ulmoides* seeds have differential gene expressions at different stages of their germination process.

The genes of samples at different stages were compared pairwise based on differences in expression levels. That is, they were divided into three groups: DZ2 vs. DZ5, DZ2 vs. DZ3, and DZ5 vs. DZ3 (Figure 5c) to study the differential expression of genes in *E. ulmoides* seeds at different germination stages. DEGs were screened, and differential expression analysis was carried out on the differential genes of each comparison combination using the standard |log_2_-Fold Change| >1, padj < 0.05. A total of 931 significantly expressed DEGs in DZ2 vs. DZ5 and 932 in DZ2 vs. DZ3 were identified, respectively. In DZ2 vs. DZ5, 743 up-regulated differential genes and 188 down-regulated differential genes. In DZ2 vs. DZ3, there were 672 up-regulated differential genes and 260 down-regulated differential genes. In DZ5 vs. DZ3, there were 274 significantly expressed DEGs, including 97 up-regulated differential genes and 177 down-regulated differential genes.

### 3.4. GO and KEGG enrichment of DEGs

**Figure 6.**
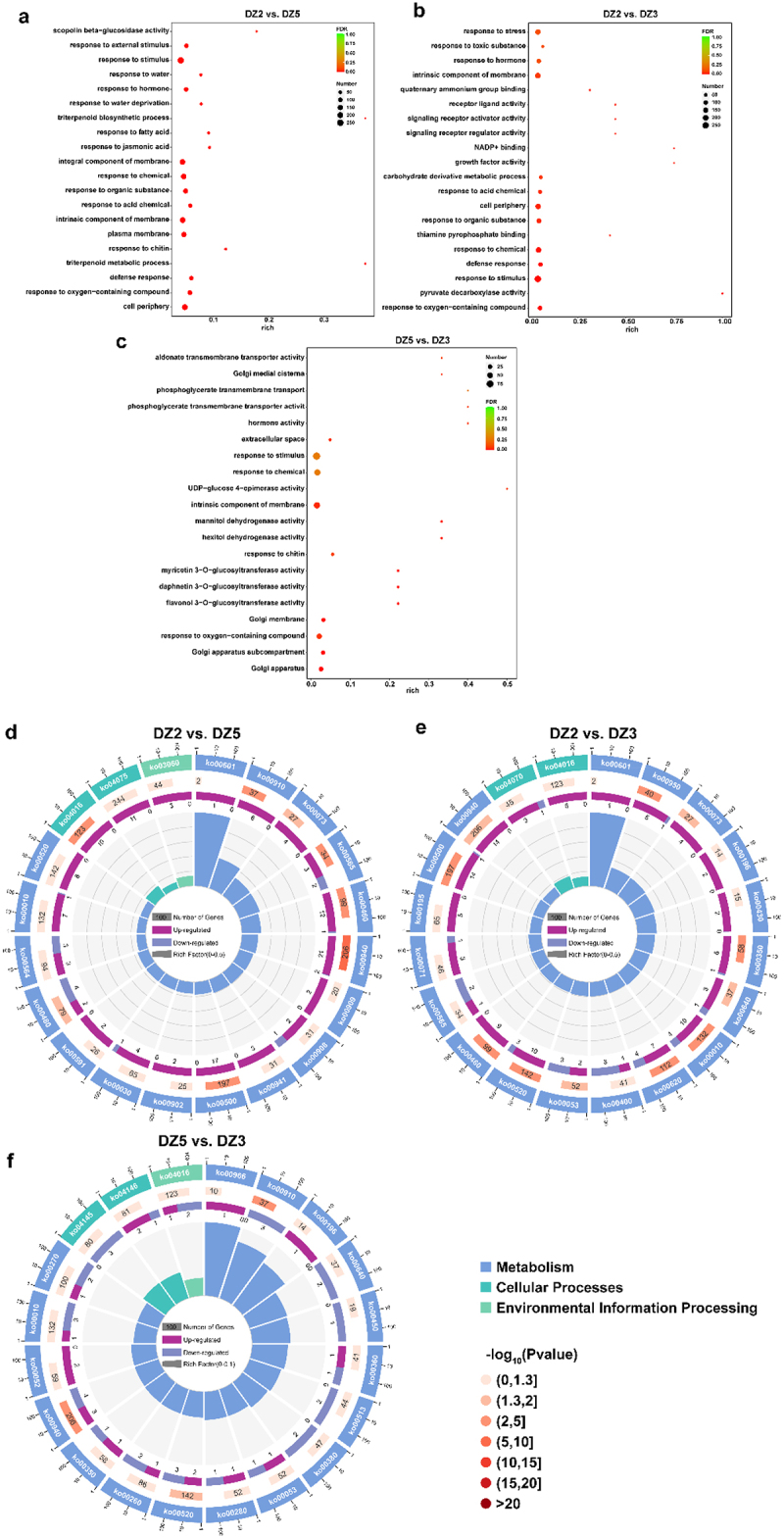
Gene ontology (Go)and Kyoto Encyclopedia of Genes and genomics enrichment (KEGG). (a-c) differentially enriched GO terms in each comparison group. (a) DZ2 vs. DZ5; (b) DZ2 vs. DZ3; and (c) DZ5 vs. DZ3. (d-f) KEGG enrichment map of differentially expressed genes in each comparison group. (d) DZ2 vs. DZ5; (e) DZ2 vs. DZ3; and (f) DZ5 vs. DZ3.

GO enrichment analysis was used to analyze the DEGs. Following the comparison of *E. ulmoides* seeds at different stages, the top 20 GO enrichment results of DEGs are shown in Figure 6(a–c). The findings show that response to stimulus (GO: 0050896) had the most enriched genes in DZ2 vs. DZ5 and DZ2 vs. DZ3. The intrinsic membrane component (GO: 0031224) has the most enriched genes in DZ5 vs. DZ3. Additionally, we discovered that response to hormone (GO: 0009725) was enriched in DZ2 vs. DZ5 and DZ2 vs. DZ3, and hormone activity (GO: 0005179) was increased in DZ5 vs. DZ3, demonstrating that hormones affect *Eucommia ulmoides* at different germination stages. Response to JA (GO: 0009753) was enriched in DZ2 vs. DZ5.

We annotated the DEGs into KEGG and performed pathway enrichment analysis to study the functions of *E. ulmoides* futher DEGs at three different stages (Figure 6d–f). The findings demonstrate that the metabolism-enriched DEGs are the most enriched among all comparison groups, while those enriched in cellular processes and environmental information processing are very few. The top 20 significantly increased KEGG pathways in DZ2 vs. DZ3 were only distributed in metabolism and cellular processes. A total of 17 KEGG pathways – phenylpropanoid biosynthesis, which was the most enriched – and 206 more DEGs were found in DZ5 vs. DZ3 in the cellular processes category. The environmental information processing category only has one enriched KEGG pathway, the MAPK signaling pathway (Figure 6f). Additionally, each comparison group had a considerably increased level of phenylpropanoid biosynthesis, suggesting that this pathway is crucial for *E. ulmoides* at different germination stages.

### 3.5. Expression of the genes for ABA、GA and IAA biosynthesis

**Figure 7.**
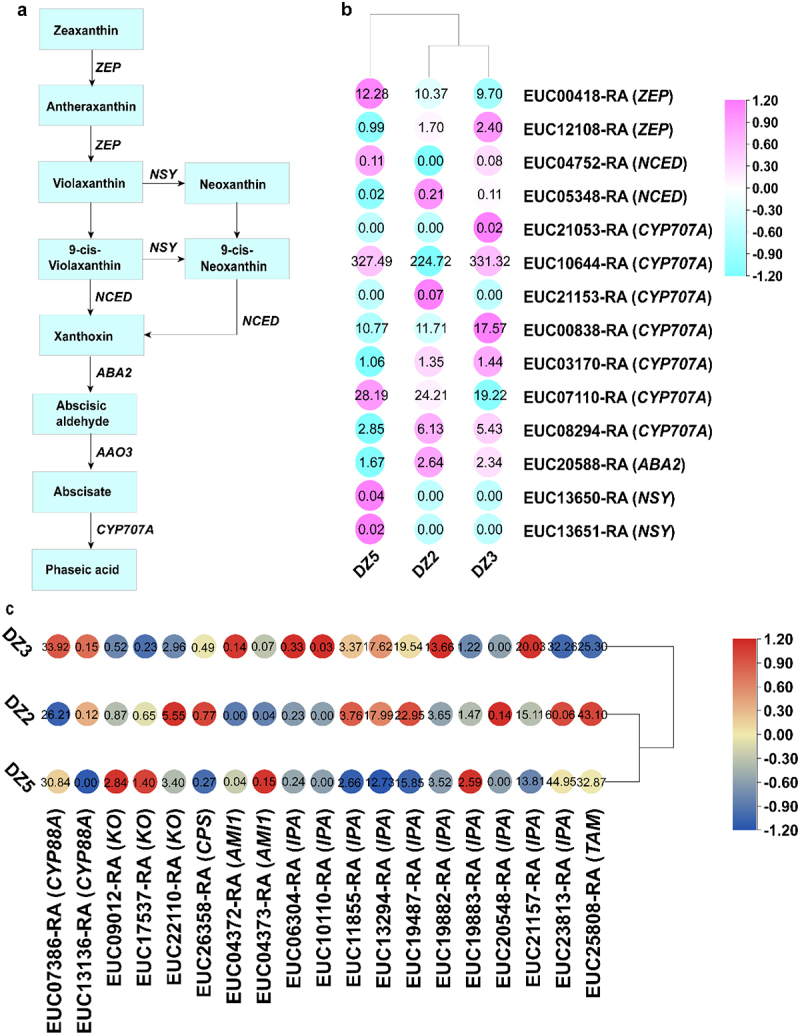
The pathways associated with abscisic acid (ABA) biosynthesis. The colored cells represent each group’s expression heatmap of the critical transcripts for ABA, GA and IAA biosynthesis. Important enzyme gene abbreviations: ZEP, zeaxanthin epoxidase; NCED, 9-cis-epoxy carotenoid dioxygenase; ABA2, xanthoxin dehydrogenase; NSY, neoxanthin synthase; KO, ent-kaurene oxidase; CPS, ent-copalyl diphosphate synthase; AMI1, amidase1; IPA, indole-3-pyruvate; TAM, tryptamine.

Zeaxanthin is the reaction substrate for ABA biosynthesis (Figure 7a). ZEP catalyzes zeaxanthin to generate antheraxanthin, which ZEP further catalyzes to develop violaxanthin. This *E. ulmoides* seed germination experiment involved a total of two ZEP gene codes. Interestingly, the expression of these two genes is entirely different. In the three stages of germination, ZEP (EUC00418-RA) showed a trend of first decreasing and then increasing. In the DZ3 stage, the expression level was the lowest; in the DZ5 stage, it was the highest. The three stages of *E. ulmoides* seeds exhibited a pattern of ZEP (EUC12108-RA) initially increasing and then decreasing. The DZ3 stage experienced the highest expression level, while the DZ5 stage had the lowest. There are two ways for violaxanthin to produce 9-cis-meoxanthin: first, it produces 9-cis-violaxanthin, which NSY then catalyzes to produce 9-cis-meoxanthin; or, second, it produces meoxanthin first, which is then catalyzed by NSY to produce 9-cis-meoxanthin. Regardless of the method, we discovered that NSY expression was 0 at the DZ2 and DZ3 stages and increased at DZ5.

9-cis-violaxanthin and 9-cis-meoxanthin both generate Xanthoxin under the catalysis of NCED, and two genes are involved in encoding NCED. The DZ2 stage accumulation of NCED (EUC04752-RA) is the lowest and gradually increases in the DZ3 and DZ5 stages. In contrast, NCED (EUC05348-RA) has the highest collection in the DZ2 stage and progressively decreases in the DZ3 and DZ5 stages. Next, Xanthoxin produces abscisic aldehyde with the catalysis of ABA2. At the DZ2 stage, ABA2 expression is at its peak. It rapidly decreases at the DZ3 and DZ5 stages, and ABA2 is eventually devoured. AAO3 catalyzes the conversion of abscisic aldehyde to abscisate, which CYP707A catalyzes to produce phaseic acid. The coding of CYP707A is distributed among a total of seven genes. During the three germination stages of *E. ulmoides* seeds, CYP707A (EUC21053-RA, EUC10644-RA, EUC00838-RA, and EUC03170-RA) showed a trend of first increasing and then decreasing. At the DZ3 stage, CYP707A (EUC21153-RA, EUC08294-RA) had the highest expression level. *E. ulmoides* seeds decreased in all three germination stages, with the DZ2 stage exhibiting the highest expression level. CYP707A (EUC07110-RA) showed a trend of first decreasing and then increasing during the three germination stages of *E. ulmoides* seeds, with the highest expression level at the DZ5 stage (Figure 7b).

The genes involved in the biosynthesis of GA and IAA mainly include CYP88A (EUC07386-RA, EUC13136-RA), KO (EUC09012-RA, EUC17537-RA, and EUC22110-RA), CPS (EUC26358-RA), AMI1 (EUC04372-RA, EUC04373-RA), IPA (EUC06304-RA, EUC10110-RA, EUC11855-RA, EUC13294-RA, EUC19487-RA, EUC19882-RA, EUC19883-RA, EUC20548-RA, EUC21157-RA, and EUC23813-RA), and TAM (EUC25808-RA) (Figure 7c). CYP88A (EUC07386-RA, EUC13136-RA) showed a trend of first increasing and then decreasing during the three germination stages of *E. ulmoides* seeds, with the highest expression level at the DZ3 stage. In the three germination stages of *E. ulmoides* seeds, KO (EUC09012-RA, EUC17537-RA, and EUC22110-RA) showed a trend of first decreasing and then increasing, with the lowest accumulation in the DZ3 stage, and KO (EUC09012-RA, EUC17537-RA) in the DZ5 stage. The accumulation amount is the highest, and KO (EUC22110-RA) has the highest amount in the DZ2 stage. Throughout the three germination stages of *E. ulmoides* seeds, CPS (EUC26358-RA) showed a gradually decreasing trend. AMI1 (EUC04372-RA) showed a trend of first increasing and then decreasing during the three germination stages of *E. ulmoides* seeds, with the highest expression level occurring at the DZ3 stage. In comparison, the expression level of AMI1 (EUC04373-RA) showed a gradually increasing trend during the three germination stages of *Eucommia* seeds. IPA (EUC06304-RA, EUC10110-RA, EUC19882-RA, and EUC21157-RA) showed a trend of first increasing and then decreasing during the three germination stages of *E. ulmoides* seeds, with the highest accumulation in the DZ3 stage. IPA (EUC11855-RA, EUC13294-RA, EUC19487-RA, and EUC20548-RA) showed a decreasing trend during the three germination stages of *E. ulmoides* seeds, and the accumulation amount was the highest in the DZ2 stage. IPA (EUC19883-RA, EUC23813-RA) showed a trend of first decreasing and then increasing during the three germination stages of *E. ulmoides* seeds. The accumulation amount was the lowest in the DZ3 stage.

### 3.6. qRT-PCR analysis

**Figure 8.**
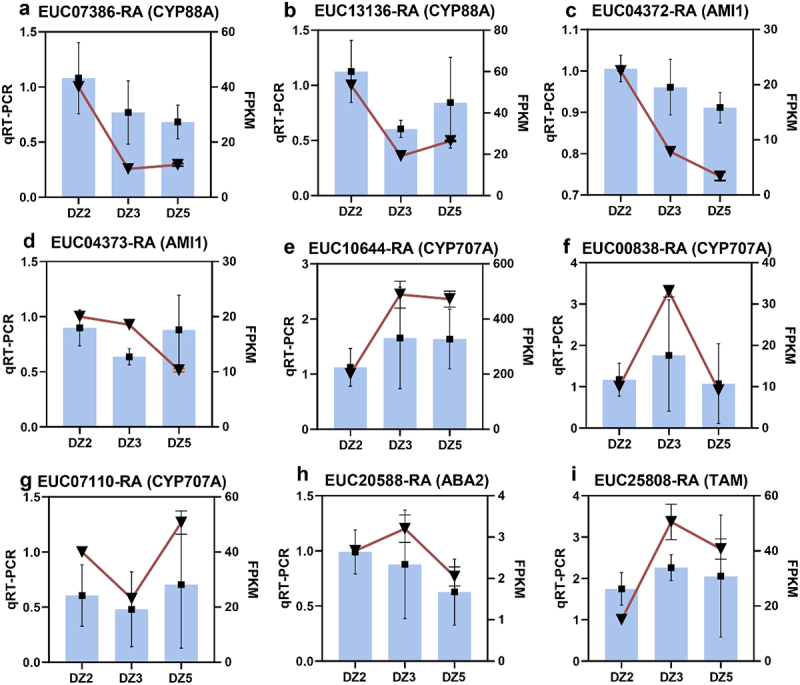
Verification of the expression patterns of RNA-seq results using qRT-PCR.

As shown in Figure 8, we selected nine genes that regulate the seed maturation process for qRT-PCR experiments. Next, we analyzed the three different genes of these nine genes in DZ2, DZ3, and DZ5 to confirm the accuracy of the transcriptome data sequencing results. Expression patterns in *E. ulmoides* seeds at the germination stage showed that EUC13136-RA (CYP707A), EUC04372-RA (AMI1), EUC10644-RA (CYP707A), EUC00838-RA (CYP707A), EUC07110-RA (CYP707A), and EUC25808 (TAM) expression pattern is consistent with the transcriptome sequencing results, and that the transcriptome sequencing results are accurate.

## 4. Discussion

The results showed that ABA, GA and IAA were the key hormones in the regulation of seed germination by analyzing the changes of plant hormone contents in different stages of seed germination. In addition, we found in the transcriptome GO and KEGG enrichment analysis that plant hormone pathways were significantly enriched at different stages of germination of Eucommia seeds, which further confirmed that plant hormones played an important role in the germination process of *E. ulmoides* seeds. We selected three phases DZ2, DZ3 and DZ5 for transcriptomic analysis in order to find the possible key genes.

The storage substances in seeds are the source of energy for maintaining seed vitality and seed germination and other life activities. Differences in the content and quality of storage substances may affect species germination,^37^ and different storage substances play different roles in the seed germination process.^38^ Soluble polysaccharide is the main functional substance of seeds in plants. Its content reflects the strength of internal metabolism of seeds.^39^ Soluble polysaccharide is directly utilized as a product of respiratory metabolism to provide energy for the development process of seeds.^37^ The soluble sugar content of *E. ulmoides* seeds is the highest in the DZ1 period. The sugar content is high in the early stage of seed germination to ensure the later germination of the seeds. The sugar content is reduced to the lowest in DZ3. At this time, the seeds are basically Germination takes shape and begins to enter the late stage of germination. Sugar plants begin to accumulate again to provide energy for seed germination, and the content increases again in the DZ5 period. Starch is mainly carbohydrate, which mainly exists in tissue cells and provides energy for the seed development process by converting into sugar.^40,41^ In this study, the accumulation of amylopectin was very high in the DZ1 period, but dropped rapidly in the DZ2 period. The possible explanation is that amylopectin converts sugar to provide energy. From the results, it can be seen that the amylose content increased from DZ1 to DZ2. It is the accumulation of starch in the early stages of seed germination that provides energy for subsequent development. However, the amylose content decreased from DZ2 to DZ4, while the amylopectin content remained basically unchanged. This may be because amylose synthesizes amylopectin under the combined action of starch synthase and coenzyme Q. The reason why amylose increased again in the DZ5 period may be that the new shoots of *E. ulmoides* seeds began to carry out photosynthesis and the starch content increased. During the germination process of *E. ulmoides* seeds, from DZ1 to DZ4, the changing trends of cellulose and cellulase contents were completely opposite. This shows that cellulase hydrolyzes cellulose and converts it into sugar, which provides energy for seed germination. From DZ4 to DZ5, the content of cellulase and cellulose increased again because the new shoots of *E. ulmoides* seeds began to undergo photosynthesis during DZ5, and photosynthesis produced glucose, which was dehydrated and polymerized to form cellulose.

ABA has the effect of inhibiting germination, inducing and maintaining seed dormancy.^23^ Therefore, reducing ABA concentration is a prerequisite for breaking seed dormancy.^42^ After seed dormancy is broken, ABA levels gradually decrease and germination begins.^43,44^ Studies have shown that after seed dormancy is released, the ABA decomposition rate is higher than the ABA synthesis rate, which promotes seed germination.^45^ This study observed *E. ulmoides* seeds at different germination stages. The results showed that the ABA content showed a trend of first increasing, then decreasing, and then increasing. The content was very high in DZ2 and DZ5 stages. Studies have pointed out that abscisic acid inhibits seed germination.^46,47^ In addition, ABA is also involved in the absorption of internal water and fertilizer in plants.^48^ We believe that the reason for the high concentration of abscisic acid may be that the seeds are in urgent need of sufficient nutrients and water to maintain normal cell metabolism, so the concentration increased twice. Seed germination is closely related to ABA biosynthesis. The ZEP gene regulates the production of ABA. Some studies on Arabidopsis thaliana have shown that expressing ZEP can increase ABA levels and delay dormancy.^49^ NCED is also an important rate-limiting enzyme in the ABA synthesis pathway. For example, overexpression of NCED in tobacco and tomato delays seed development and inhibits seed germination.^50,51^ In our study, NCED (EUC05348-RA) expressed the highest amount at the DZ3 stage, and then its accumulation decreased steadily. From this, we speculated that it is a key gene in the ABA synthesis pathway in Eucommia seeds. A crucial step in the synthesis of Phaseic acid is the generation of Abscisate under the catalysis of the CYP707A gene. The CYP707A family gene is one of the important genes that regulates ABA production.^52^ There are many reports about CYP707A family genes. Saito et al. found in their study of dormant and non-dormant seeds of Arabidopsis that AtCY707A2 gene expression plays a dominant role in ABA content.^53^ Matilla et al. found that AtCYP707A2 is a key gene that regulates ABA levels in Arabidopsis seeds and promotes seed germination.^52^ Another study confirmed that the protein encoded by the CYP707A2 gene is involved in the ABA anabolism process in the mutant and promotes its germination by reducing the ABA content in the mutant.^54^ There are 7 gene segments involved in encoding the ABA synthesis pathway in *E. ulmoides* seed germination. Among them, CYP707A2 (EUC21053-RA, EUC10644-RA, EUC00838-RA and EUC03170-RA) is involved in the *E. ulmoides* seed germination. The concentration is the highest in DZ3 and then decreases. This is consistent with the above the results.

GA plays a very important role in promoting seed germination.^55^ In this study, we measured the hormone content of *E. ulmoides* seeds at different stages. The results showed that the GA content showed a trend of first decreasing and then increasing during the germination process of *E. ulmoides* seeds. In the DZ2 stage, the GA content of *E. ulmoides* seeds is the lowest. Too low GA content will hinder the activation of related metabolic enzymes inside the seeds. Therefore, we speculate that the reason why GA increases and reaches its peak in the DZ5 stage is that the GA content increases in the later stages of seed germination. The expression of hydrolytic enzymes in softened barrier tissue reduces the mechanical resistance of the radicle tip, which is conducive to the radicle breaking through the seed coat. However, further research is needed on the specific relationship between GA and hydrolase during the germination process of *E. ulmoides* seeds. Seed germination is regulated by the dynamic balance of ABA and GA levels.^21^ As ABA anabolism is weakened or catabolism is enhanced, ABA content is reduced, GA anabolism is increased or catabolism is weakened, and GA levels are regulated, causing dormancy to be broken and seeds to begin to germinate. Wang et al. used dormant Arabidopsis thaliana (Arabidopsis thaliana) as experimental subjects and proved that ABA levels are negatively correlated with seed germination rate.^56^ However, germination rate is affected by the synergistic effect of GA rather than a single hormone.^57^ ABI4 directly binds to the promoter region of ABA catabolism-related genes CYP707A1 and 331 CYP707A2, promoting the accumulation of ABA. ABA and GA have antagonistic effects. The expression frequency of genes involved in GA synthesis is higher, while the expression frequency of genes involved in GA catabolism is lower.^58^ Studies on rice have found that OsAP2–39 inhibits seed germination by promoting the expression of genes related to ABA synthesis and GA inactivation (such as OsNCED1), thereby increasing ABA levels and reducing GA levels.^59^ Auxin is the second hormone that promotes seed dormancy besides ABA,^26^ and its content will affect seed dormancy. Tan et al. measured the hormone content of carrot seeds at different germination stages and found that the IAA content showed a gradually increasing trend,^60^ which is consistent with the results of this study that the IAA content gradually increased from DZ1 to DZ4. Some studies have pointed out that the physiological effects of auxin are two-sided,^61^ that is, it promotes plant growth and seed germination at low concentrations, but it has a hindering effect at higher concentrations.^62^ One of the explanations for the above phenomenon may be that the high concentration in the mid-term has a certain inhibitory effect on seed germination. The inside of the seed reduces the synthesis of auxin through negative feedback regulation, thereby promoting seed germination. This explains the phenomenon that IAA content decreased again during the DZ5 period in this study. Studies have shown that ABA interacts with auxin, and ABA inhibits hypocotyl elongation by enhancing auxin signals.^26^ The mechanism of action of IAA and ABA during *E. ulmoides* seed germination is still unclear.

## 5. Conclusions

We determined the physiological and hormonal indexes of the seeds at different stages of germination. We concluded that soluble sugar was the main energy supply substance during the germination of the seeds, while amylose, amylopectin and cellulose provided energy by transforming into soluble sugar. We found that the contents of abscisic acid (ABA), gibberellin (GA) and auxin (IAA) changed significantly with seed germination, suggesting that these three hormones were closely related to seed germination. In order to further study, we used transcriptome sequencing technology to study the differentially expressed genes at three different stages of *E. ulmoides* seed germination, and initially revealed the dynamic changes of differentially expressed genes at the transcriptome level during the germination of Eucommia seeds. The hormone-related synthesis pathway was found in the control group of Eucommia seeds at different stages of germination, which further confirmed this conclusion. In addition, in our study, we speculated that NCED and CYP707A are important genes in the ABA synthesis pathway of *E. ulmoides* seeds. However, the mechanism of ABA, GA and IAA in the germination process of *E. ulmoides* seeds remains unclear and needs further study.
